# Furin Egress from the TGN is Regulated by Membrane‐Associated RING‐CH Finger (MARCHF) Proteins and Ubiquitin‐Specific Protease 32 (USP32) via Nondegradable K33‐Polyubiquitination

**DOI:** 10.1002/advs.202403732

**Published:** 2024-07-19

**Authors:** Wenqiang Su, Iqbal Ahmad, You Wu, Lijie Tang, Ilyas Khan, Bowei Ye, Jie Liang, Sunan Li, Yong‐Hui Zheng

**Affiliations:** ^1^ State Key Laboratory for Animal Disease Control and Prevention Harbin Veterinary Research Institute Chinese Academy of Agricultural Sciences Harbin China; ^2^ Department of Preventive Veterinary Medicine College of Veterinary Medicine Northeast Agricultural University Harbin Heilongjiang China; ^3^ Center for Bioinformatics and Quantitative Biology Richard and Loan Hill Department of Biomedical Engineering The University of Illinois Chicago Chicago IL 60607 USA; ^4^ Department of Microbiology and Immunology The University of Illinois Chicago Chicago IL 60612 USA

**Keywords:** furin, K33‐polyubiquitination, MARCHF, post‐Golgi, USP32

## Abstract

Furin primarily localizes to the *trans*‐Golgi network (TGN), where it cleaves and activates a broad range of immature proproteins that play critical roles in cellular homeostasis, disease progression, and infection. Furin is retrieved from endosomes to the TGN after being phosphorylated, but it is still unclear how furin exits the TGN to initiate the post‐Golgi trafficking and how its activity is regulated in the TGN. Here three membrane‐associated RING‐CH finger (MARCHF) proteins (2, 8, 9) are identified as furin E3 ubiquitin ligases, which catalyze furin K33‐polyubiquitination. Polyubiquitination prevents furin from maturation by blocking its ectodomain cleavage inside cells but promotes its egress from the TGN and shedding. Further ubiquitin‐specific protease 32 (USP32) is identified as the furin deubiquitinase in the TGN that counteracts the MARCHF inhibitory activity on furin. Thus, the furin post‐Golgi trafficking is regulated by an interplay between polyubiquitination and phosphorylation. Polyubiquitination is required for furin anterograde transport but inhibits its proprotein convertase activity, and phosphorylation is required for furin retrograde transport to produce fully active furin inside cells.

## Graphical abstract

MARCHF E3 ubiquitin ligases (M2, M8, M9) polyubiquitinate furin via K33‐linkage, which inhibits furin activity in the TGN but promotes its egress from the TGN. However, this process is counteracted by a deubiquitinase USP32. These findings fill in an important gap in understanding the full furin trafficking cycle, which is known to be partially regulated by phosphorylation.

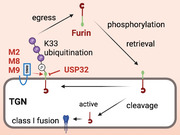

## 1 Introduction

The proprotein convertase subtilisin/kexin type (PCSK) family consists of 9 serine proteases that post‐translationally cleave immature precursor proteins to trigger activation and play physiologically essential functions.^[^ ^1^ ^]^ Furin/PCSK3 is the founding member of this family, which targets numerous mammalian, viral, and bacterial substrates. The mammalian substrates include more than 100 cytokines, hormones, growth factors, and receptors, and aberrant furin expression or activation has been associated with cancer and other developmental and neuronal diseases.^[^ ^2^ ^]^ Viral envelope glycoproteins from at least 13 different virus families comprise another significant category of furin substrates.^[^ ^3^ ^]^ For example, furin cleaves class I fusion proteins, including those from highly pathogenic human viruses, such as Ebola virus (EBOV) glycoprotein (GP), human immunodeficiency virus type 1 (HIV‐1) envelope glycoprotein (Env), severe acute respiratory syndrome coronavirus 2 (SARS‐CoV‐2) spike protein (S), and influenza A virus hemagglutinin protein (HA).^[^ ^4^ ^]^ Thus, furin is a promising therapeutic target for noninfectious and infectious human diseases.

Human furin is a 794‐amino acid (aa) type I transmembrane (TM) protein containing a signal peptide (SP), prodomain (Pro), subtilisin‐like catalytic domain (CD), P‐domain, cysteine‐rich region (CRR), TM domain, and cytoplasmic tail (CT).^[^ ^5^ ^]^ Furin cleaves a basic amino acid motif R‐X‐(R/K/X)‐Arg↓ that is also present twice in its prodomain. Furin self‐cleaves its prodomain that acts as an intermolecular chaperone to promote its folding in the ER and trafficking to the Golgi. Furin is then completely activated after further cleavage in CRR by a poorly defined mechanism. Fully active furin primarily localizes to the *trans*‐Golgi network (TGN) where it cleaves and activates proproteins. The TGN localization of furin is determined by its 56‐aa CT via a retrieval mechanism rather than TGN retention. The CT membrane‐proximal region has a dileucine‐based (^756^LI^757^) and a tyrosine‐based (^759^YKGL^762^) endocytic sorting motif that direct furin from the cell surface to early endosomes and subsequently to late endosomes.^[^ ^6^ ^]^ Downstream of these sorting motifs is an acidic cluster ^769^EECPSDSEEDE^779^, in which S773 and S775 are phosphorylated by casein kinase II (CKII).^[^ ^7^ ^]^ Phosphorylated furin binds phosphofurin acidic cluster sorting protein‐1 (PACS‐1) which also binds the adaptor protein 1 (AP1) complex,^[^ ^8^ ^]^ and allows AP1 to retrieve furin from late endosomes to the TGN.^[^ ^9^ ^]^ This late endosome‐TGN retrieval pathway also requires the GTPase Rab9.^[^ ^10^ ^]^ Phosphorylated furin can also be dephosphorylated by protein phosphatase 2A (PP2A), which retrieves furin from early endosomes to the TGN.^[^ ^11^ ^]^ During maturation, furin is subjected to ectodomain cleavage in CRR to become fully activated by an unknown cellular endoprotease and undergoes shedding.^[^ ^12^ ^]^ The cleavage primarily occurs at the N‐terminal to R683, but there is an alternative cleavage site very close to the C‐terminal to R683.^[^ ^12^ ^]^ Nevertheless, little is known about how furin exits the TGN for anterograde trafficking.

Like phosphorylation, ubiquitination is another reversible post‐translational modification (PTM) that plays a critical role in the regulation of cellular processes.^[^ ^13^ ^]^ Ubiquitination not only controls protein turnover via proteasomes and lysosomes but also governs a variety of cellular signaling pathways via nondegradable mechanisms. Ubiquitin (Ub) chains are assembled with 8 structurally and functionally distinct polymers via the seven lysine residues (K6, K11, K27, K29, K33, K48, K63), or the N‐terminal methionine (M1) of Ub. This process is catalyzed by an E1 Ub‐activating enzyme, an E2 Ub‐conjugating enzyme, and an E3 Ub ligase. The substrate specificity of ubiquitination is determined by the large number of E3s. RING (Really Interesting New Gene) E3s are the most abundant Ub ligases, which include the membrane‐associated RING‐CH finger (MARCHF) family proteins. The MARCHF family consists of 11 members, and except for MARCHF7 and MARCHF10, they all have at least two TM domains.^[^ ^14^ ^]^ Most of them function as immune regulators that polyubiquitinate the CT of immune receptors and downregulate them by lysosomal degradation. Recently, we and others reported that MARCHF8 inhibits the replication of a wide range of enveloped viruses by targeting their fusion proteins.^[^ ^15^ ^]^ Notably, MARCHF8 inhibits the maturation of class I fusion proteins in the TGN by blocking the furin cleavage, *N*‐glycosylation, and *O*‐glycosylation.^[^ ^15^, ^16^ ^]^ This nondegradable inhibitory mechanism does not depend on any lysine residues in the class I fusion CT but requires the RING domain of MARCHF proteins, which remains poorly understood.

Ubiquitination is a strictly regulated process that can be reversed by deubiquitinases (DUBs).^[^ ^17^ ^]^ They are cysteine proteases or metalloproteases that cleave peptide or isopeptide bonds between Ub molecules or between Ub and substrate proteins. DUBs often directly bind specific targets via protein‐interactive domains distinct from the catalytic domain, and they also select targets by recognizing Ub chain architectures. The human genome encodes ≈100 DUBs, which are divided into seven families. The largest family comprises the Ub‐specific proteases (USPs), which are cysteine proteases with ≈60 members. Many USPs have clear subcellular localizations expressed in different cellular compartments and organelles.

Here, we identify MARCHF2 (M2), MARCHF8 (M8), and MARCHF9 (M9) as the E3 Ub ligases that polyubiquitinate the furin CT via K33‐linked Ub chains, which is required for furin egress from the TGN. We further identified USP32 as the furin DUB that counteracts the furin polyubiquitination by M2, M8, and M9. These findings provide new insights into the poorly defined furin post‐Golgi trafficking mechanism, which has important implications for the potential development of furin‐based therapies.

## 2 Results

### 2.1 Effect of MARCHF Proteins on EBOV‐GP Expression

**Figure 1:**
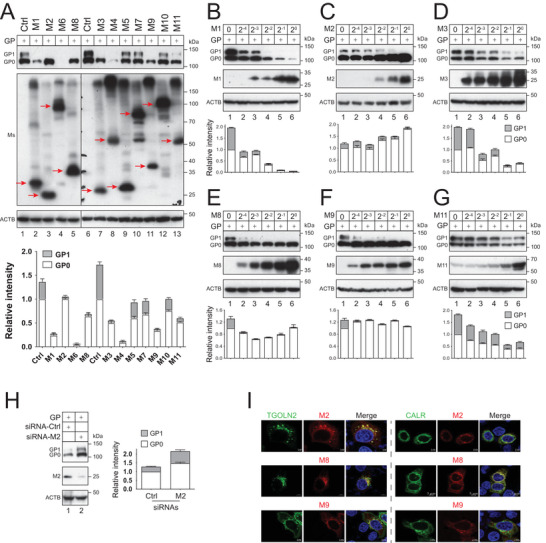
Effect of MARCHF proteins on EBOV‐GP protein expression. A) EBOV‐GP with a FLAG‐tag was expressed with M1–M11 with a HA‐tag in HEK293T cells. Protein expression was determined by WB. MARCHF proteins are indicated by a red arrow. B–G) EBOV‐GP was expressed with increasing amounts of M1, M2, M3, M8, M9, and M11 expression vectors in 6‐well plates, and their protein expression was determined by WB. The undiluted vectors (2^0^) were used as 1 µg per well. H) EBOV‐GP was expressed with *M2*‐specific or scrambled (control, Ctrl) small interference RNAs (siRNA) in HeLa cells. Protein expression was determined by WB. I) M2, M8, and M9 with a mCherry‐tag were expressed with TGOLN2 or CALR with an eGFP‐tag in HeLa cells. After being stained with DAPI, fluorescent signals were detected by confocal microscopy (scale bar 5 µm). The intensity of GP_1_ and GP_0_ bands in (A)–(H) were quantified by ImageJ, and their relative levels are presented. Error bars indicate standard error of measurements (SEMs) calculated from three experiments (*n* = 3). All experiments were repeated at least three times, with similar results obtained.

To understand how the MARCHF family proteins affect EBOV‐GP expression, eleven MARCHF proteins (M1–M11) were expressed with EBOV‐GP in HEK293T cells, and their expression was detected by Western blotting (WB). All these MARCHF proteins were expressed at a comparable level, but they differentially affected EBOV‐GP expression. EBOV‐GP is heavily glycosylated in its mucin‐like domain (MLD) during maturation in the Golgi, resulting in a much higher molecular mass of the GP_1_ subunit than the unprocessed GP_0_ precursor (**Figure** **1**A, lanes 1, 6), as we reported.^[^ ^18^ ^]^ M5, M7, and M10 did not affect GP_0_ or GP_1_ protein expression (Figure 1A, lanes 9, 10, 12), whereas M4 and M6 reduced both GP_0_ and GP_1_ protein expression (Figure 1A, lanes 4, 8). However, M1, M2, M3, M8, M9, and M11 seemed to selectively decrease the GP_1_ protein expression (Figure 1A, lanes 2, 3, 5, 7, 11, 13). To confirm this selective activity, EBOV‐GP was expressed with serially diluted M1, M2, M3, M8, M9, and M11, and GP expression was determined. M1, M3, and M11 decreased both GP_0_ and GP_1_ protein expression (Figure 1B,D,G), whereas only M2, M8, and M9 selectively decreased the GP_1_ protein expression in a dose‐dependent manner (Figure 1C,E,F). This selectively inhibitory activity was detected at 1/16 dilution (62.5 ng DNA input per well in a 6‐well tissue culture plate), which was less than *M8* transcripts achieved upon type I interferon stimulation at early time points.^[^ ^15a^ ^]^ These results suggest that M2, M8, and M9 could inhibit the EBOV‐GP proteolytic cleavage at physiologically relevant protein expression levels.

Because MARCHF proteins attack many cell surface proteins, their expression is tightly regulated, which causes their poor expression in immortalized cell lines.^[^ ^19^ ^]^ Although we could not detect M8 and M9 protein expression, we confirmed a weak M2 expression in HeLa cells by WB as reported previously.^[^ ^20^ ^]^ When the M2 expression was silenced by small interference RNAs (siRNA), we detected an increase of GP_1_ protein expression in HeLa cells (Figure 1H). This result confirmed that the endogenous M2 protein inhibits the EBOV‐GP proteolytic cleavage.

Because GP undergoes proteolytic cleavage in the TGN, we investigated the subcellular localization of M2, M8, and M9 using calreticulin (CALR) and *trans*‐Golgi network integral membrane protein 2 (TGOLN2) as the ER or TGN marker, respectively. Although M8 was also found in the ER, they were all found in the TGN (Figure 1I). Thus, we decided to focus on M2, M8, and M9 to understand how they block EBOV‐GP proteolytic processing in the TGN.

### 2.2 Mechanistic Analysis of M2, M8, and M9 Inhibition of EBOV‐GP Processing

**Figure 2:**
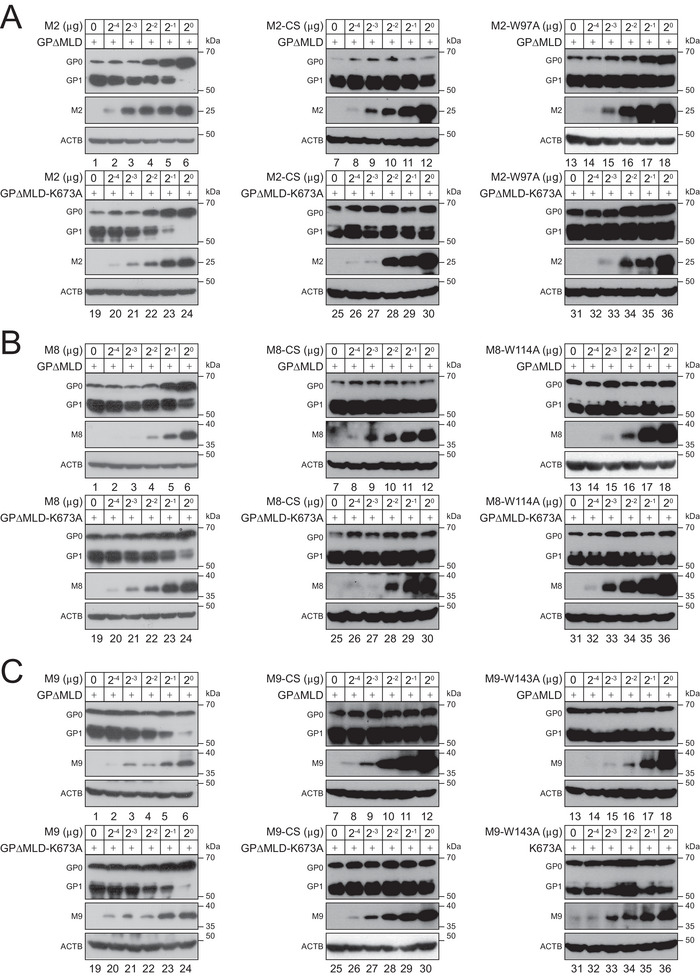
Mechanistic analysis of M2, M8, and M9 inhibition of EBOV‐GP processing. A) EBOV‐GP∆MLD and its K673 mutant with a FLAG‐tag were expressed with increasing amounts of M2 or its two catalytically inactive mutants (CS, W97A) in HEK293T cells, respectively, and EBOV‐GP proteolytic processing was determined by WB. B,C) Similar experiments were done with M8 and its two mutants (CS, W114A), and M9 and its two mutants (CS, W143A), and results are shown in (B) or (C). All experiments were repeated at least three times, with similar results obtained.

We and others reported that two M8 RING domain mutants CS and W114A do not have any antiviral activity.^[^ ^15a,b,d^ ^]^ The M8 CS mutant consists of four C‐to‐S substations (C80S/C83S/C123S/C126S), and these critical cysteine residues are also conserved in M2 (C64/C67/C106/C109) or M9 (C110/C113/C152/C155). The M8 W114 residue is also conserved in M2 (W97) or M9 (W143). Thus, analogous RING domain mutants were created for M2 and M9. We reported that EBOV‐GP with MLD‐deletion (GP∆MLD) is proteolytically processed much more efficiently in cells, resulting in much higher virus particle infectivity.^[^ ^15^, ^18^, ^21^ ^]^ Thus, GP∆MLD was used to detect the EBOV‐GP proteolytic processing in the following experiments. When M2, M8, M9, and their RING domain mutants were expressed with GP∆MLD, only the wild‐type (WT) proteins blocked GP processing in a dose‐dependent manner, whereas all the mutants did not (**Figure** **2**A–C, lanes 1–18), confirming that RING domain that governs the E3 Ub ligase activity is required for the inhibitory activity.

EBOV‐GP CT has a single lysine residue K673. When K673 was mutated to alanine (K673A), M2, M8, and M9 still blocked the GP processing, whereas their RING domain mutants did not (Figure 2A–C, lanes 19–36). These results confirm that these MARCHF proteins do not target K673 to block the EBOV‐GP processing, as reported previously.^[^ ^15a^ ^]^

### 2.3 Broadness of M2, M8, and M9 Activity

**Figure 3:**
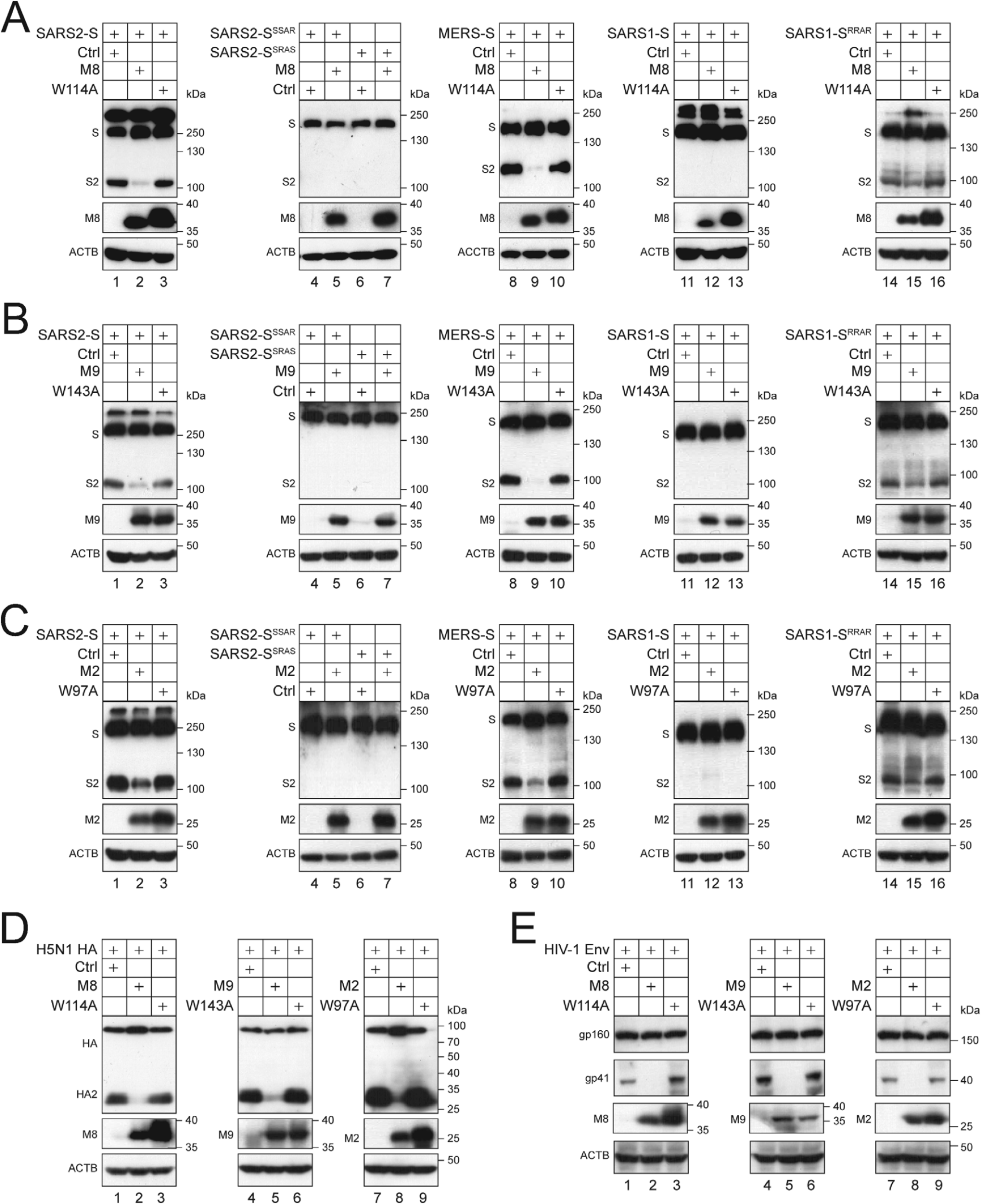
Broadness of M2, M8, and M9 activity. A) Class I fusion proteins and mutants were expressed with M8 or W114A in HEK293T cells. Their proteolytic processing was determined by WB. B,C) Similar experiments were performed with M9 or W143A, and M2 or W97A, and results are shown in (B) and (C). M2, M8, and M9 were further tested on H5N1 HA and HIV‐1 Env, and D,E) results are shown. All experiments were repeated at least three times, with similar results obtained.

SARS2‐S, MERS‐S, influenza A virus H5N1 HA, and HIV‐1 Env are also proteolytically processed by furin, whereas SARS1‐S is not. We created SARS2‐S furin‐cleavage defective mutants SARS2‐S^SSAR^ and SARS2‐S^SRAS^, and similarly, we also created a furin‐cleavage site in SARS1‐S by constructing a mutant SARS1‐S^RRAR^. When SARS2‐S, SARS2‐S^SSAR^, SARS2‐S^SRAS^, MERS‐S, SARS1‐S, and SARS1‐S^RRAR^ were expressed with M8 or its W114A mutant in HEK293T cells, the proteolytic cleavage of SARS2‐S, MERS‐S, and SARS1‐S^RRAR^ was blocked by M8, but not W114A (**Figure** **3**A, lanes 1–3, 8–10, 14–16). No cleavage product was detected from SARS2‐S^SSAR^, SARS2‐S^SRAS^, and SARS1‐S, and M8 did not affect their expression (Figure 3A, lanes 4–7, 11–13). Similar results were collected from experiments with M9 or W143A mutant (Figure 3B), and M2 or W97A mutant (Figure 3C). M8, M9, and M2 also blocked the proteolytic processing of H5N1 HA (Figure 3D, lanes 2, 5, 8) and HIV‐1 Env (Figure 3E, lanes 2, 5, 8), whereas their W114A, W143A, or W97A mutant did not (Figure 3D,E, lanes 3, 6, 9). Collectively, these results demonstrate that M2, M8, and M9 broadly block the proteolytic processing of class I fusion proteins.

### 2.4 M2, M8, and M9 directly Inhibit Furin

**Figure 4:**
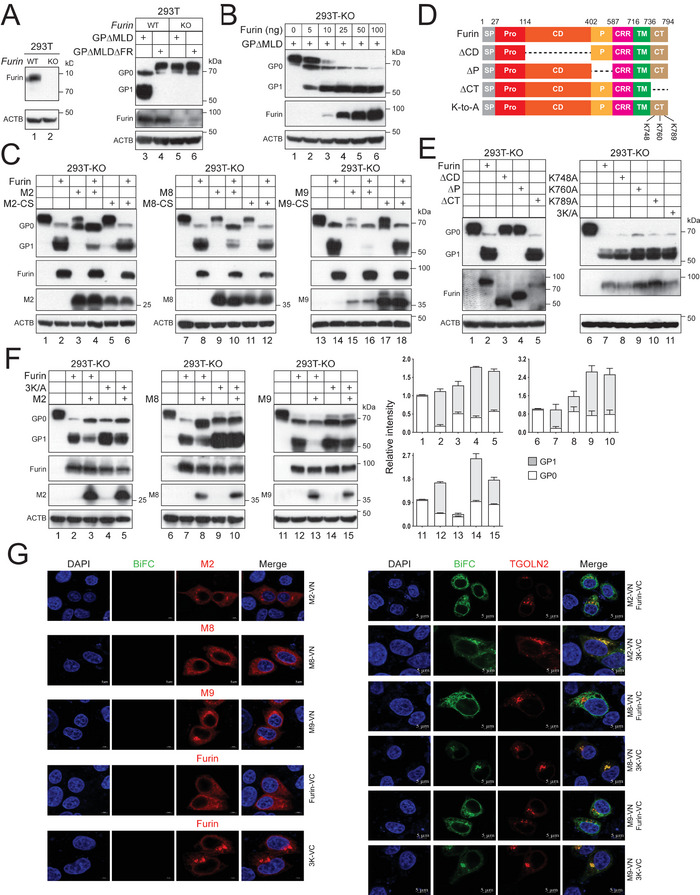
M2, M8, and M9 inhibit furin directly. A) *Furin* was knocked out in HEK293T cells by CRISPR/Cas9, and its protein expression in wild‐type (WT) and knockout (KO) cells was confirmed by WB. GP∆MLD and its mutant ∆FR were expressed in these cells, and their proteolytic processing was determined by WB. B) Increasing amounts of furin were expressed with GP∆MLD in *furin*‐KO cells, and furin activity was measured by WB. C) GP∆MLD was expressed with MARCHF and furin in *furin*‐KO cells, and GP processing was determined by WB. D) A schematic representation of furin and mutants generated. SP, signal peptide; Pro, prodomain; CD, subtilisin‐like catalytic domain; P, P‐domain; CRR, cysteine‐rich region; TM, transmembrane domain; CT, cytoplasmic tail. Numbers indicate aa positions. Three CT lysine (K) residues are indicated. E) Furin WT and mutants were expressed with GP∆MLD in *furin*‐KO cells, and GP processing was determined by WB. F) GP∆MLD was expressed with indicated furin and MARCHF in *furin*‐KO cells, and GP processing was determined by WB. The GP_1_ and GP_0_ expression levels were quantified by ImageJ and are presented as relative values. Error bars indicate SEMs calculated from three experiments (*n* = 3). G) MARCHF with a VN‐tag and furin or 3K/A with a VC‐tag were expressed alone, and cells were stained with DAPI and anti‐HA for MARCHF or anti‐FLAG for furin. In addition, these MARCHF proteins were expressed with these furin proteins and TGOLN2 with a mCherry‐tag in HeLa cells. Green BiFC signals and MARCHF, furin, and TGOLN2 expression were detected by confocal microscopy (scale bar 5 µm). All experiments were repeated at least three times, with similar results obtained.

To measure the furin activity, we knocked out *furin* in HEK293T cells by CRISPR/Cas9 and created a *furin*‐knockout (KO) cell line (**Figure** **4**A, lanes 1, 2). When GP∆MLD and its furin‐cleavage defective mutant (∆FR) were expressed in WT or KO cells, GP_1_ was detected from GP∆MLD in WT cells, but not in KO cells, and no cleavage product was detected from ∆FR (Figure 4A, lanes 3–6). We then expressed GP∆MLD with furin in KO cells and could detect the GP cleavage when as few as 5 ng furin expression vector was used (Figure 4B). These results demonstrate that furin cleaves EBOV‐GP very effectively in cells. Thus, we established a very sensitive cell‐based assay to measure furin activity. Importantly, we found that M2, M8, and M9 inhibited furin activity, whereas their RING domain CS mutants did not (Figure 4C).

To understand how furin is targeted by these MARCHF proteins, we created three furin deletion mutants, ∆CD, ∆P, and ∆CT by deleting CD, P‐domain, or CT, respectively (Figure 4D). Furin has three lysine residues (K748, K760, K789) in CT, which were also mutated to alanine individually (K748A, K760A, K789A) or together (3K/A). When these furin mutants were expressed with GP∆MLD in *furin*‐KO cells, ∆CT and four lysine mutants still actively cleaved this viral protein, but ∆CD and ∆P did not (Figure 4E). These results demonstrate that furin does not require its CT, particularly the three lysine residues, to cleave EBOV‐GP, whereas its CD and P‐domain are required.

Next, we investigated how these MARCHF proteins target furin. When GP∆MLD was expressed with furin in the presence of MARCHF proteins in *furin*‐KO cells, M2, M8, and M9 inhibited the cleavage activity of WT furin, but not 3K/A (Figure 4F). These results suggested that MARCHF proteins likely target furin via these lysine residues in CT. We then used bimolecular fluorescence complementation (BiFC) assay to track the furin‐MARCHF interaction, as we did previously.^[^ ^22^ ^]^ M2, M8, and M9 with a VN‐tag were expressed in HeLa cells with furin or 3K/A with a VC‐tag, and TGOLN2 with a mCherry‐tag. Green BiFC signals were detected in these cells, only when furin was expressed with M2, M8, or M9, indicating that these MARCHF proteins interact with furin and 3K/A in live cells (Figure 4G). However, BiFC signals from these MARCHF proteins with furin did not colocalize with TGOLN2, whereas those with 3K/A did (Figure 4G). These results demonstrate that M2, M8, and M9 relocate furin from the TGN to the other compartments via the CT lysine residues.

### 2.5 M2, M8, and M9 Polyubiquitinate Furin CT via K33‐linked Ub Chains

**Figure 5:**
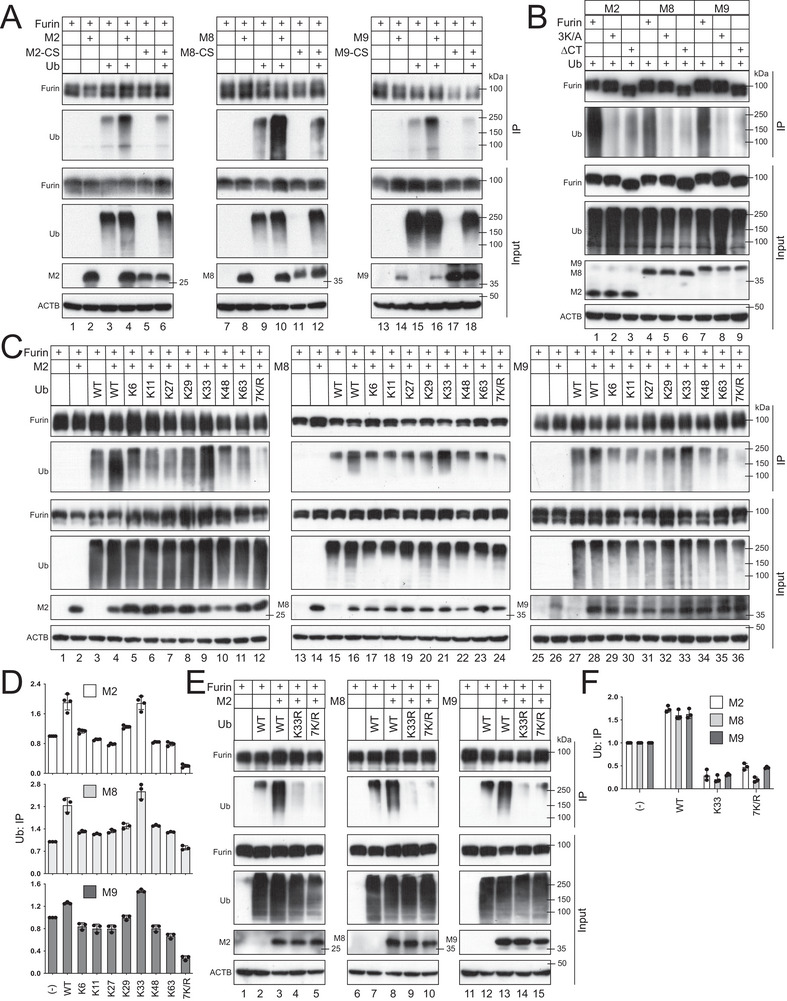
M2, M8, and M9 polyubiquitinate furin CT via K33‐linked Ub chains. A) FLAG‐tagged furin was expressed with MARCHF and His‐tagged Ub proteins in HEK293T cells. Proteins were immunoprecipitated (IP) with anti‐FLAG, and furin polyubiquitination was analyzed by WB. B) Furin WT and mutants were expressed with MARCHF and Ub proteins in HEK293T cells. Furin polyubiquitination was detected by WB similarly. C) Ub WT and mutants only expressing single K or none of any K (7K/R) were expressed with furin and MARCHF proteins in HEK293T cells. Furin polyubiquitination was detected by WB. D) The intensity of Ub bands from IP in (C) was quantified by ImageJ and presented as relative values. Error bars indicate SEMs calculated from three or four experiments (*n* = 3 or 4). E) Ub WT, K33R, and 7K/R were expressed with furin and MARCHF proteins in HEK293T cells. Furin polyubiquitination was detected by WB. F) The intensity of Ub bands from IP in (E) was quantified by ImageJ and presented as relative values. Error bars indicate SEMs calculated from three experiments (*n* = 3).

To understand whether K748, K760, and K789 are polyubiquitinated, M2, M8, or M9 were expressed with furin in the presence of ectopic Ub, and furin polyubiquitination was detected by immunoprecipitation (IP). All these MARCHF proteins strongly promoted furin polyubiquitination, whereas their CS mutants did not (**Figure** **5**A). However, they did not promote the polyubiquitination of 3K/A and ∆CT (Figure 5B). These results demonstrate that these MARCHF proteins polyubiquitinate furin at residues K748, K760, and K789, although we could not eliminate the possibility that they might also target another polyubiquitinated protein that binds furin via these lysine residues.

We then determined the Ub chain structure. Initially, we used seven Ub mutants, K6, K11, K27, K29, K33, K48, and K63, that only expressed one of the respective seven lysine residues. In addition, we included another Ub mutant 7K/R, in which all seven lysine residues were mutated to arginine (R). M2, M8, and M9 promoted furin polyubiquitination only in the presence of Ub WT and K33 (Figure 5C, lanes 4, 9, 16, 21, 28, 33, the IP panels; Figure 5D). These results suggested that the K33 residue plays an important role in furin polyubiquitination. To validate this conclusion, we repeated this experiment with another Ub mutant K33R, in which only the K33 residue was mutated to arginine. Strong furin polyubiquitination was detected in the presence of WT Ub, but not K33R and 7K/R (Figure 5E, the IP panel; Figure 5F). Collectively, these results confirm the important role of the K33‐linkage in furin polyubiquitination.

### 2.6 M2, M8, and M9 Promote Furin Anterograde Trafficking to the Plasma Membrane

**Figure 6:**
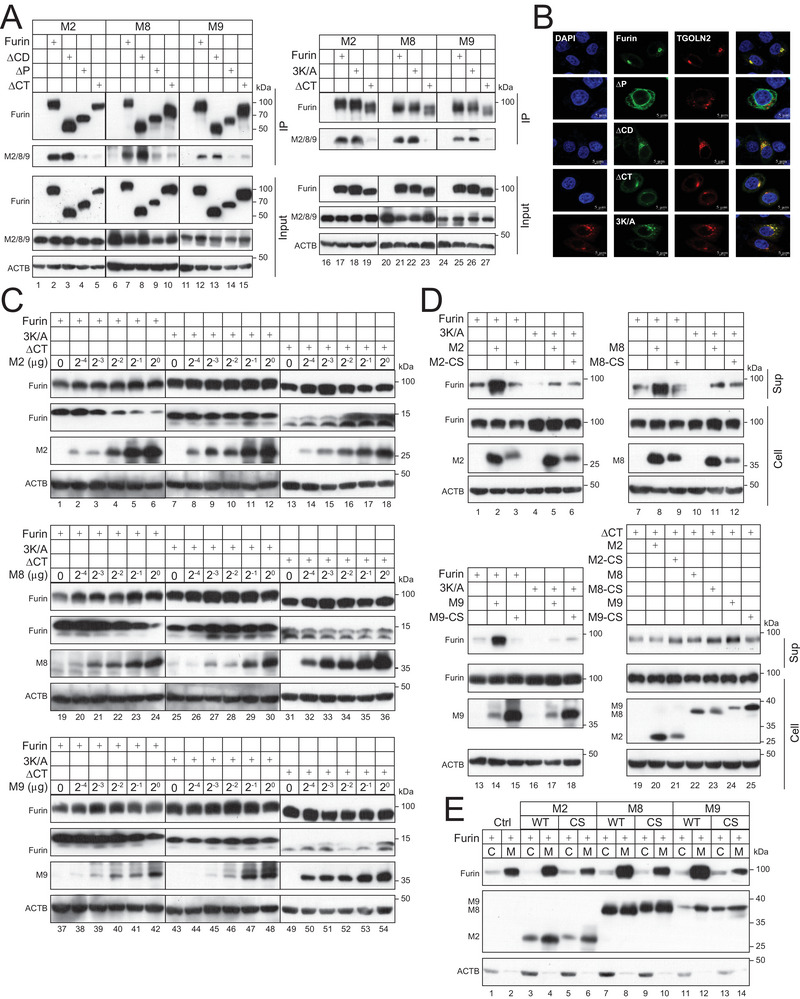
M2, M8, and M9 promote furin shedding. A) FLAG‐tagged furin WT and mutants were expressed with HA‐tagged MARCHF proteins in HEK293T cells. Proteins were immunoprecipitated (IP) with anti‐FLAG and analyzed by WB. B) eGFP‐tagged furin WT and mutants were expressed with mCherry‐tagged TGOLN2 in HeLa cells. After staining with DAPI, eGFP, and mCherry expression were determined by confocal microscopy (scale bar 5 µm). C) Furin WT and mutants were expressed with increasing amounts of MARCHF proteins in HEK293T cells. Furin self‐processing was determined by WB. D) FLAG‐tagged furin WT and mutants were expressed with MARCHF proteins in HEK293T cells. Furin proteins were pulled down from the supernatants with anti‐FLAG, and levels of shed furin were determined by WB. E) FLAG‐tagged furin was expressed with MARCHF WT and CS mutant in HEK293T cells. Cells were fractionated and furin expression in the plasma membrane (M) and cytosolic (C) factions were determined by WB. All experiments were repeated at least three times, with similar results obtained.

To understand where furin is targeted from the TGN by MARCHF proteins, we investigated the furin intracellular trafficking. Initially, we tried to map the MARCHF‐binding domain on furin by IP. When furin WT, ∆CD, ∆P, ∆CT, and 3K/A were expressed with M2, M8, and M9, only furin, ∆CD, and 3K/A could pull down these MARCHF proteins, whereas ∆P and ∆CT could not. (**Figure** **6**A). To understand whether both P‐domain and CT could serve as the MARCHF binding surface, we determined the subcellular localization of these furin mutants. We found that unlike furin, ∆CD, ∆CT, and 3K/A, ∆P showed a cytoplasmic distribution that no longer colocalized with TGOLN2 (Figure 6B), indicating that ∆P no longer localizes to the TGN. Thus, M2, M8, and M9 should not bind P‐domain, rather, they bind CT to polyubiquitinate furin. In addition, K748, K760, and K789 are not required for this binding.

Furin becomes fully activated after cleavage in CRR.^[^ ^12^ ^]^ To understand how M2, M8, and M9 affect this cleavage process, furin WT, 3K/A, and ∆CT were expressed with increasing amounts of MARCHF in HEK293T cells. We could detect one strong band at ≈15 kD from furin and 3K/A, which is the cleavage product from the primary site R683 (Figure 6C, lanes 1–12, 19–30, 37–48). A weak lower band was also detected from these proteins, which is the product from the alternative cleavage site. These two small bands were also detected from ∆CT at much reduced molecular weights as expected but at similar levels. Importantly, M2, M8, and M9 reduced the levels of these small bands from furin in a dose‐dependent manner, but not 3K/A and ∆CT (Figure 6C). Thus, M2, M8, and M9 inhibit this furin maturation by polyubiquitination.

Furin undergoes ectodomain shedding after maturation.^[^ ^12^ ^]^ We then determined how M2, M8, and M9 affect furin shedding. Furin WT, ∆CT, and 3K/A were expressed with these MARCHF and their CS mutants. Furin proteins were pulled down from the supernatants and the levels of shed furin were determined by WB. M2, M8, and M9 strongly increased furin shedding whereas their CS mutants did not (Figure 6D, lanes 2, 3, 8, 9, 14, 15). However, this activity was significantly compromised when 3K/A and ∆CT were used (Figure 6D, lanes 5, 6, 11, 12, 17, 18, 19–25). To confirm that furin is targeted to the cell surface, we purified the plasma membrane from cells expressing furin and MARCHF proteins, as we did previously.^[^ ^23^ ^]^ M2, M8, and M9 strongly increased furin expression in the plasma membrane fraction (M) but not in the cytosolic fraction (C), whereas their CS mutants did not have this activity (Figure 6E). Collectively, these results demonstrate that M2, M8, and M9 promote furin trafficking to the cell surface and shedding by targeting the CT lysine residues.

### 2.7 Mapping of Furin‐Binding Domain on MARCHF Proteins

**Figure 7:**
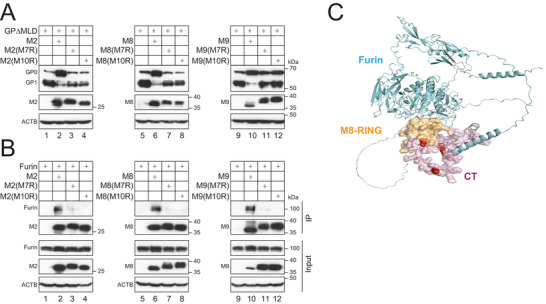
Mapping of furin‐binding domain on MARCHF. A) GP∆MLD was expressed with MARCHF RING domain mutants in HEK293T cells. GP processing was determined by WB. B) HA‐tagged MARCHF RING domain mutants were expressed with FLAG‐tagged furin in HEK293T cells. Proteins were immunoprecipitated (IP) with anti‐HA and analyzed by WB. C) The RING domain (aa 72–133, light orange) from a partial M8 structure (aa 1–157, UNIPROT: P09958) is predicted to interact with furin CT (aa 736–794, light pink) from a furin structure (aa 1–794, UNIPROT: P0DTC2). K748, K760, and K789 are highlighted in red. All experiments were repeated at least three times, with similar results obtained.

We showed that the RING domain mutants did not block the furin activity, suggesting that the RING domain is required for the MARCHF activity. We swapped the RING domain in M2, M8, and M9 with those from M7 and M10 that do not inhibit EBOV‐GP cleavage (Figure 1A). These chimeric proteins are named M2(M7R), M2(M10R), M8(M7R), M8(M10R), M9(M7R), and M9(M10R). None of these mutants blocked EBOV‐GP processing (**Figure** **7**A). We then determined their interaction with furin by IP and found that they also lost binding to furin (Figure 7B).

Human M2, M8, and M9 consist of 246, 291, or 346 aa, respectively, and have a RING domain in the N‐terminal CT, a linker (L) domain, two TM domains, and a C‐terminal CT (Figure S1A, top panels, Supporting Information). We created M2, M8, and M9 deletion mutants by deleting the N‐terminal domain (∆N), RING domain (∆R), L domain (∆L), and C‐terminal domain (∆C). When these mutants were expressed with GP∆MLD in HEK293T cells, only ∆N inhibited the GP proteolytic processing, whereas the other mutants did not (Figure S1A, Supporting Information). These results demonstrate that all these domains, except for the N‐terminal domain, are required for MARCHF activity. In addition, like the full‐length protein, ∆N, ∆L, and ∆C pulled down furin, whereas ∆R did not (Figure S1B, Supporting Information). These results further confirm that M2, M8, and M9 bind furin via their RING domain.

The furin CT in a predicted structure (UNIPROT/AF2: P09958) features a random‐coil‐like configuration (Figure S2A,B, Supporting Information). The predicted M8 RING domain structure (UNIPROT/AF2: P0DTC2) consists of alpha‐helices and random‐coil or loop regions^[^ ^24^ ^]^ that are crucial for protein‐protein interactions and the E3 Ub ligase activity (Figure S2C,D, Supporting Information). Based on five sets of finely‐tuned hyperparameters by default in the ColabFold server,^[^ ^25^ ^]^ five predicted complexes of the furin‐M8 RING domain were obtained using AlphaFold2‐Multimer.^[^ ^26^ ^]^ In four instances, we detected a direct interaction between RING and furin CT using alphashape^[^ ^27^ ^]^ (Figure 7C), indicating a robust signal for a consistent binding pattern within the complex of the furin‐M8 RING domain. Notably, in three out of these four predicted structures, K789 was found in the binding interface. Both furin and M8 sequences exhibit a reliable depth of multiple sequence alignment (over 100) (Figure S3A, Supporting Information). In addition, the predicted local‐distance difference test (pLDDT) scores for the interacting region of RING (aa 72–133) and furin CT (aa 736–794) are approximately or exceed a threshold of 70, indicating high levels of confidence in predictions of these residues (Figure S3B, Supporting Information). The specific interface residues are summarized in Table S1 in the Supporting Information.

### 2.8 Identification of USP32 as Furin Deubiquitinase (DUB)

**Figure 8:**
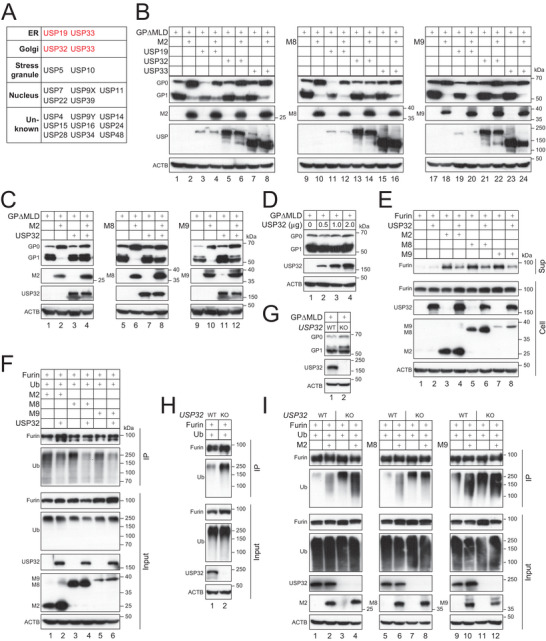
Identification of USP32 as furin deubiquitinase (DUB). A) Furin with a FLAG‐tag was expressed in HEK293T cells. Proteins were immunoprecipitated with anti‐FLAG and analyzed by mass spectrometry. Identified DUBs and their cellular localization are shown. B) DUBs were expressed with GP∆MLD and MARCHF proteins in HEK293T cells. GP processing was determined by WB. C) USP32 was expressed with MARCHF and GP∆MLD in HEK293T cells. GP processing was determined by WB. D) GPΔMLD was expressed with increasing USP32 in HEK293T cells. GP processing was determined by WB. E) Furin was expressed with USP32 and MARCHF proteins in HEK293T cells. Furin shedding was determined by WB. F) FLAG‐tagged furin was expressed with MARCHF and His‐tagged Ub proteins in the presence or absence of ectopic USP32 in HEK293T cells. Furin polyubiquitination was determined by WB after pulldown by anti‐FLAG. G) GP∆MLD was expressed in HEK293T WT and *USP32*‐KO cells, and GP processing was determined by WB. H) FLAG‐tagged furin was expressed with HA‐tagged Ub in HEK293T WT and *USP32*‐KO cells, and furin polyubiquitination was determined by WB after pulldown by anti‐FLAG. I) FLAG‐tagged furin was expressed with MARCHF and HA‐tagged Ub in HEK293T WT and *USP32*‐KO cells. Furin polyubiquitination was determined by WB after pulldown by anti‐FLAG. All experiments were repeated at least three times, with similar results obtained.

The human genome encodes more than 100 DUBs.^[^ ^28^ ^]^ They are divided into six major subclasses that have different subcellular localizations.^[^ ^29^ ^]^ We pulled down furin from HEK293T cells and analyzed its protein complex by mass spectrometry. We identified 19 DUBs from this complex, and three of them (USP19, USP32, USP33) are expressed in the ER and/or Golgi (**Figure** **8**A). When they were expressed with MARCHF and GP∆MLD in HEK293T cells, USP32 increased the GP_1_ protein levels, whereas USP19 and USP33 did not (Figure 8B, lanes 6, 14, 22). This USP32 activity was further confirmed by another independent experiment (Figure 8C, lanes 4, 8, 12). In addition, USP32 alone did not affect the GP∆MLD processing (Figure 8D). Considering that USP32 is expressed in the Golgi, these results suggest that USP32 could specifically target furin.

To validate the USP32 activity, we determined how USP32 affects furin shedding. When furin and USP32 were expressed with M2, M8, and M9, these MARCHF proteins increased furin shedding, which was inhibited by USP32 (Figure 8E, lanes 4, 6, 8). Next, we determined how USP32 affects furin polyubiquitination. Ectopic USP32 expression strongly inhibited the furin polyubiquitination in the presence of M2, M8, and M9 (Figure 8F, lanes 2, 4, 6).

To further confirm the USP32 activity, we created a *USP32*‐KO cell line from HEK293T cells by CRISPR/Cas9. When GP∆MLD was expressed, we detected a slight decrease in GP_1_ but an increase in GP_0_ protein expression in KO cells compared to WT cells (Figure 8G), likely due to an enhancement of the endogenous MARCHF activity. Consistently, the furin polyubiquitination was also significantly increased in KO cells than WT cells (Figure 8H). Furthermore, when ectopic M2, M8, and M9 were expressed, they also induced much stronger furin polyubiquitination in KO cells than WT cells (Figure 8I). Collectively, these results demonstrate that USP32 is the furin DUB that counteracts the M2, M8, and M9 activity.

### 2.9 USP32 USP Domain Binds Furin CT

**Figure 9:**
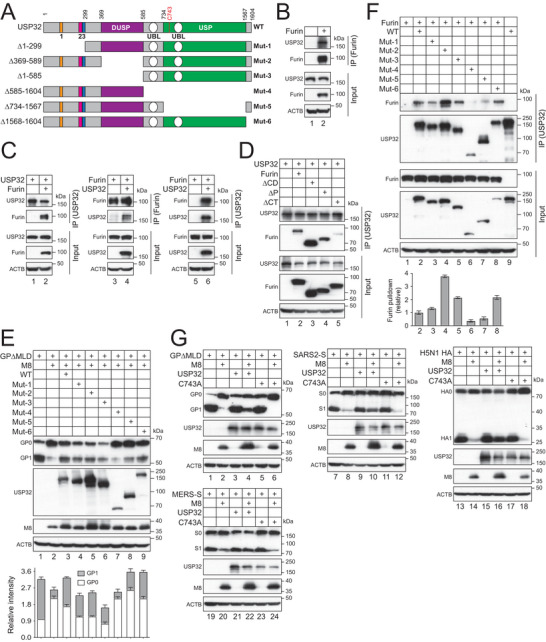
USP32 USP domain binds furin CT. A) A schematic representation of USP32 WT and deletion mutants. USP, Ub‐specific protease; DUSP, domain in USP; UBL, Ub‐like; 1, 2, 3, calcium‐binding EF‐hand 1, 2, and 3. B) FLAG‐tagged furin was expressed in HEK293T cells and proteins were immunoprecipitated (IP) with anti‐FLAG. USP32 was detected with a specific antibody by WB. C) Myc‐tagged USP32 was expressed with FLAG‐tagged furin in HEK293T cells. Proteins were immunoprecipitated with anti‐Myc (lanes 1, 2, 5, 6) or anti‐FLAG (lanes 3, 4) and detected by WB. D) HA‐tagged USP32 was expressed with furin mutants in HEK293T cells. Proteins were immunoprecipitated with anti‐HA and detected by WB. E) GP∆MLD and M8 were expressed with USP32 mutants in HEK293T cells. GP processing was determined by WB. Levels of GP_1_ and GP_0_ were quantified by ImageJ and their relative levels are shown at the bottom panel. F) FLAG‐tagged furin was expressed with HA‐tagged USP32 mutants. Proteins were immunoprecipitated with anti‐HA and analyzed by WB. Levels of furin from the pulldown were quantified using ImageJ and are shown as relative values at the bottom panel. G) USP32 WT and C743A were expressed with class I fusion proteins in the presence or absence of M8 in HEK293T cells. Their proteolytic processing was analyzed by WB. Error bars in (E) and (F) indicate SEMs calculated from three experiments (*n* = 3). All experiments were repeated at least three times, with similar results obtained. All experiments were repeated at least three times, with similar results obtained.

Human USP32 has 1604 aa, which consists of a C‐terminal USP domain, two Ub‐like (UBL) domains, one domain in USP (DUSP), and three calcium‐binding EF‐hands 1, 2, 3 (**Figure** **9**A). Initially, we found that ectopic furin could pull down the endogenous USP32 in HEK293T cells by IP (Figure 9B). Next, we expressed both furin and USP32 in HEK293T cells and found that they could pull down each other reciprocally (Figure 9C). These results confirmed the furin‐USP32 binding. To map the USP32‐binding domain on furin, USP32 was expressed with furin WT, ∆CD, ∆P, and ∆CT in HEK293T cells. USP32 could pull down furin, ∆P, and ∆CD, but not ∆CT (Figure 9D). These results demonstrate that USP32 binds furin CT.

To map the furin‐binding domain on USP32, we created six USP32 deletion mutants (Mut‐1 to 6) (Figure 9A). When these mutants were expressed with M8 and GP∆MLD, only Mut‐4 and Mut‐5 did not significantly recover the GP_1_ levels that were decreased by M8 (Figure 9E, compare lanes 7 and 8 with 2). In addition, only these two mutants did not pull down furin (Figure 9F, lanes 6, 7, IP panel). Because neither Mut‐4 nor Mut‐5 has the USP domain, these results demonstrate that the USP domain is required for the furin binding.

C743 is the principal catalytic residue of USP32 (Figure 9A).^[^ ^30^ ^]^ To further validate the USP32 function, we mutated C743 to alanine (C743A) and tested its activity. Class I fusion proteins including EBOV‐GP, SARS2‐S, H5N1 HA, and MERS‐S were expressed with M8 and WT USP32 or C743A in HEK293T cells, and their proteolytic processing was determined. The cleavage of these viral glycoproteins was blocked by M8 (Figure 9G, lanes 2, 8, 14, 20). This cleavage inhibition was counteracted by USP32 WT but not C743A (Figure 9G, lanes 4, 6, 10, 12, 16, 18, 22, 24). Collectively, these results confirm that the USP domain plays a very critical role in USP32 deubiquitination of furin.

## 3 Discussion

**Figure 10:**
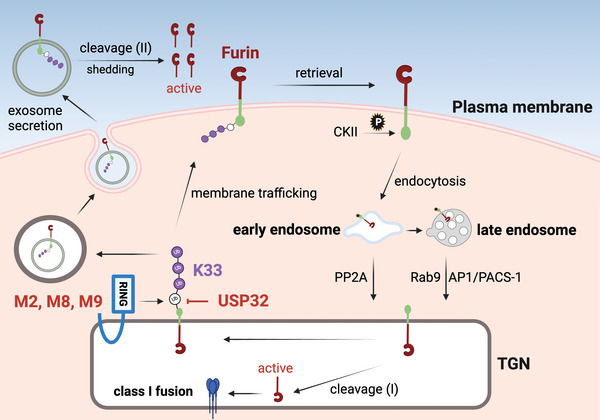
A Model for Furin Post‐Golgi Trafficking. After removal of the prodomain, furin is subjected to K33‐polyubiquitination by M2, M8, and M9 to egress the TGN, which is counteracted by USP32. Polyubiquitinated furin undergoes exocytosis via exosomes, where it is cleaved by an unknown cellular protease for shedding, producing extracellularly active fuin. Polyubiquitinated furin also migrates to the cell surface, which is phosphorylated by CKII for endocytosis. Phosphorylated furin undergoes retrograde transport from early endosomes to late endosomes, where it binds PACS1 and is retrieved to the TGN by AP1 and Rab9. Furin is also dephosphorylated by PP2A, which directly retrieves furin from early endosomes to the TGN. Retrieved furin is re‐targeted by M2, M8, and M9 for the next round of cycling. Alternatively, furin is activated by another unknown cellular protease in the TGN to produce intracellularly active furin that processes proproteins such as class I fusion proteins for maturation.

From investigation on inhibition of the proteolytic processing of class I fusion glycoproteins, we identify M2, M8, and M9 as the furin E3 Ub ligases. They localize in the TGN in which they bind furin CT and trigger its K33‐polyubiquitination, which promotes furin egress from the TGN and transport it to the cell surface. We further describe USP32 as the furin DUB, which inhibits this anterograde transport by counteracting M2, M8, and M9. These findings fill an important gap in our understanding of furin post‐Golgi trafficking and regulation of its activity (**Figure** **10**).

Phosphorylation plays a fundamental role in furin retrograde transport to the TGN, but it has been unclear how furin exits the TGN to initiate anterograde transport.^[^ ^5^ ^]^ We now demonstrate that this trafficking process is controlled by polyubiquitination of its K748, K760, and K789 (Figure 10). Previously, the tyrosine‐based motif ^759^YKGL^762^ in furin CT was found to be required for furin egress from the TGN,^[^ ^9^ ^]^ suggesting that AP1 is involved in this process. However, the furin mutant used in that investigation had a CT deletion after S758, resulting in loss of K760 and K789. Thus, the deficiency in furin trafficking could have been due to poor polyubiquitination of this mutant. Generally, K33‐polyubiquitination is a nondegradable PTM that has been implicated in post‐Golgi transport.^[^ ^23^, ^31^ ^]^ Using a sensitive BiFC assay, it was found that K33‐polyubiquitination chains are recruited to sequestosome 1 (SQSTM1/p62), and this process is modulated by ZRANB1/TRABID,^[^ ^32^ ^]^ which functions as a K29‐ and K33‐specific DUB.^[^ ^33^ ^]^ SQSTM1/p62 is a Ub‐binding protein that functions as an autophagy adaptor but also has many other functions.^[^ ^34^ ^]^ For example, SQSTM1/p62 directly interacts with the motor protein dynein and is required for dynein motility and trafficking along microtubules.^[^ ^35^ ^]^ In addition, SQSTM1/p62 is subjected to K29‐ and K33‐polyubiquitination by RNF166, which is required for the adaptor function.^[^ ^36^ ^]^ Thus, we speculate that SQSTM1/p62 may play a role in furin K33‐mediated post‐Golgi trafficking.

Furin undergoes ectodomain shedding after cleavage in CRR, resulting in the release of ≈15‐kDa fragment. We now find that M2, M8, and M9 inhibit this furin cleavage and block its maturation. Although we detected a decrease in the 15‐kDa fragment production by these MARCHF proteins, we did not detect a change in the full‐length furin expression (Figure 6C). These results suggest that only a small portion of furin is subjected to this cleavage. Guanylate‐binding protein (GBP) 2 and 5, which are GTPases hydrolyzing GTP to GDP and GMP, were reported to inhibit furin.^[^ ^37^ ^]^ Similarly, GBP2 and GBP5 decreased the 15‐kDa fragment production but did not change the full‐length furin expression.^[^ ^37^ ^]^ Nevertheless, we also find that M2, M8, and M9 strongly promote furin shedding. These results provide new insights into the regulation of furin post‐Golgi trafficking and its proprotease activity. We speculate that after egress from the TGN, polyubiquitinated furin may undergo exocytosis via exosomes, where it is cleaved by an unknown cellular protease for shedding, producing extracellularly active fuin. Such exosome release mechanism was reported in CD171 shedding after cleavage by ADAM17 and ADAM10.^[^ ^38^ ^]^ Alternatively, polyubiquitinated furin may also migrate to the cell surface and is phosphorylated by CKII for endocytosis, resulting in furin retrieval from early and late endosomes into the TGN, where it is retargeted by M2, M8, and M9 for the next round of cycling. However, furin can also be activated by another unknown cellular protease, producing intracellularly active furin to process proproteins such as class I fusion proteins for maturation (Figure 10). Thus, polyubiquitination escorts furin for anterograde transport but inhibits its intracellular activity, whereas phosphorylation is required for furin retrograde transport to the TGN, where it can be fully activated to cleave proproteins. It is unclear why furin requires such a sophisticated mechanism to regulate its post‐Golgi trafficking and activity. Probably, its intracellular activity needs to be fine‐tuned to avoid any aberrant activation that may cause various diseases.

We find that M2, M8, and M9 promote furin ectodomain shedding, which should be due to their inhibition of the furin endocytosis via polyubiquitination. The furin K760 is located within the AP2‐binding tyrosine‐based motif ^759^YKGL^762^ in CT, and thus its polyubiquitination may disrupt AP2 binding to this motif. In addition, it was reported that the E3 Ub ligases CBLB and ITCH mediate T‐cell receptor z‐chain K33‐polyubiquitination, which inhibits its phosphorylation by ZAP70.^[^ ^39^ ^]^ In the case of furin, K748, K760, and K789 are very close to S773 and S775 that are phosphorylated by CKII. Furin polyubiquitination may also inhibit its phosphorylation. Thus, polyubiquitination may stabilize the levels of furin on the cell surface, which enhances furin trafficking to the exocytosis pathway by a feedback mechanism, resulting in increase of furin shedding from exosomes (Figure 10).

We demonstrated that M2, M8, and M9 directly inhibit furin after ectopic expression of furin with EBOV‐GP in *furin*‐KO cells (Figure 4A). In addition to furin/PCSK3, EBOV‐GP could also be processed by PCSK5 and PCSK7.^[^ ^40^ ^]^ It was surprising that the proteolytic cleavage of EBOV‐GP was completely blocked in the absence furin, suggesting that these KO cells may not express PCSK5 and PCSK7. In addition, we found that the ∆P mutant lost binding to M2, M8, and M9 (Figure 6A), which is consistent with a recent report.^[^ ^16a^ ^]^ However, we also found that this mutant is no longer expressed in the TGN (Figure 6B). Importantly, this P‐domain is on the ectodomain of furin, which makes it unlikely to approach the cytoplasmic RING domain of these MARCHF proteins. Thus, M2, M8, and M9 should bind furin CT, but not its P‐domain, to inhibit its activity. It was also shown that MARCHF1 (M1) could inhibit the furin activity.^[^ ^16a^ ^]^ However, we found that M1 decreased both GP_1_ and GP_0_ expression in a dose‐dependent manner, suggesting that it should not specifically block furin.

We have identified USP32 as the furin DUB. USP38 has been found to specifically cleave K33‐linked Ub chains on TBK1.^[^ ^41^ ^]^ However, USP32 does not have any obvious cleavage preferences for the eight Ub linkages, indicating that it does not recognize specific Ub chain architectures.^[^ ^30^ ^]^ Consistently, we found that USP32 specifically interacts with furin CT for deubiquitination. Our results demonstrate that USP32 acts together with M2, M8, and M9 to fine‐tune the intracellular activity of furin via K33‐polyubiquitination, perhaps providing a new strategy to target furin for therapeutic development.

## 4 Experimental Section

### Cells

HEK293T cells and HeLa cells were obtained from the American Type Culture Collection. They were maintained in Dulbecco's modified Eagle medium (DMEM) supplemented with 10% fetal calf serum (FBS) and 1% penicillin–streptomycin and cultured at 37 °C with 5% CO_2_.

### Plasmids

Codon‐optimized *MARCHF1* (GenBank AB463878.1)*, MARCHF2* (GenBank AB197929.1)*, MARCHF3* (GenBank AK290129.1)*, MARCHF4* (GenBank NM_02 0814.3)*, MARCHF5* (GenBank AB191202.1)*, MARCHF6* (GenBank NM_0 05885.4)*, MARCHF7 (GenBank NM_0 012 82805.2), MARCHF9* (GenBank NM_138 396.6)*, MARCHF10* (GenBank XM_0 052 57095.4), and *MARCHF11* (GenBank NM_0 011 02562.3) were synthesized and cloned into the pCAGGS vector with a 3×HA‐tag. MARCH2, MARCH8, and MARCH9 expressing a mCherry‐tag were generated by PCR using pCAGGS‐MARCHF2‐3×HA, pCAGGS‐3×HA‐MARCHF8, and pCAGGS‐MARCHF9‐3×HA as template followed by cloning into pCAGGS‐mCherry by EcoRI digestion. The plasmids pCAGGS‐3×HA‐MARCHF8, pCAGGS‐3×HA‐MARCHF8‐W114A, pcDNA3.1‐FLAG‐EBOV‐GP, EBOV‐GPΔMLD, and EBOV‐GPΔMLDΔFR and pCMV6‐furin‐Myc‐FLAG were reported.^[^ ^15d^ ^]^ The plasmids pCAGGS‐3×HA‐MARCHF8‐W114A‐3×HA, pCAGGS‐MARCHF2‐W97A‐3×HA, and pCAGGS‐MARCHF9‐W143A‐3HA were created by site‐directed mutagenesis using pCAGGS‐MARCHF2‐3×HA or pCAGGS‐MARCHF9‐3×HA as template. To generate the RING domain CS mutants, C64S/C67S/C106S/C109S, C80S/C83S/C123S/C126S, and C110S/C113S/C152S/C155S mutations were introduced into pCAGGS‐MARCHF2‐3×Myc, pCAGGS‐3×Myc‐MARCHF8, or pCAGGS‐MARCHF9‐3×Myc, respectively. These three vectors were constructed from pCAGGS‐MARCHF2‐3×HA, pCAGGS‐3×HA‐MARCHF8, and pCAGGS‐MARCHF9‐3×HA by replacing 3×HA with 3×Myc after EcoRI/NheI digestion. The plasmid pcDNA3.1‐FLAG‐GPΔMLD‐K673A was created from pcDNA3.1‐EBOV‐GP∆MLD by site‐directed mutagenesis. pcDNA3.1‐HA‐EBOV‐GPΔMLD was created by PCR from pcDNA3.1‐FLAG‐EBOV‐GPΔMLD by BamHI/EcoRI digestion. The furin deletion mutant ΔCD (aa 114–402), ΔP (aa 402–587), and ΔCT (aa 736–794) were generated by PCR using pCMV6‐furin‐Myc‐FLAG as a template. The furin‐3K mutation was created by replacing the lysine (K) at positions 748, 760, and 789 with alanine (A). Vectors expressing eGFP‐tagged furin and its mutants (ΔCD, ΔP, ΔCT, and 3K) were generated by PCR using pCMV6‐furin‐Myc‐FLAG as template followed by cloning into pEGFP‐N1 by Kpn1/Age1 digestion. pCMV6‐FLAG‐furin‐FLAG and pCMV6‐furin‐His were created by PCR from pCMV6‐furin‐FLAG‐Myc by AsiSI/PmeI digestion. The BiFC expression vectors of pcDNA3.1‐MARCHF2‐HA‐VN, pcDNA3.1‐MARCHF8‐HA‐VN, pcDNA3.1‐MARCHF9‐HA‐VN, pcDNA3.1‐furin‐FLAG‐VC, and pcDNA3.1‐furin‐3K‐FLAG‐VC were created from pcDNA3.1‐HA‐VN or pcDNA3.1‐FLAG‐VC by XhoI/BspEI digestion.^[^ ^22^ ^]^ Ub and mutant expression vectors were reported previously.^[^ ^23^ ^]^ pCAGGS‐SARS1‐S‐FLAG, pCAGGS‐SARS2‐S‐FLAG, and pCAGGS‐MERS‐S‐FLAG were reported recently.^[^ ^42^ ^]^ pCAGGS‐SARS2‐S^SSAR^‐FLAG and pCAGGS‐SARS2‐S^SARS^‐FLAG were created by mutating the furin cleavage site ^682^RRAR^685^ to ^682^SSAR^685^ or ^682^SARS^685^. pCAGGS‐SARS1‐S^RRAR^‐FLAG was created by replacing ^663^VSLLR^667^ with QTNSPRRAR derived from SARS2‐S. The *TGOLN2* and *CALR* genes were synthesized and cloned into pEGFP‐N1 after KpnI/AgeI digestion. *TGOLN2* was also cloned into pCAGGS with a mCherry tag. pCAGGS‐H5N1 HA‐FLAG and the HIV‐1 Env expression vector were reported previously.^[^ ^21^, ^43^ ^]^

pCAGGS‐MARCHF2(M7R)−3×HA, pCAGGS‐MARCHF2(M10R)−3×HA, pCAGGS‐3×HA‐MARCHF8(M7R), pCAGGS‐3×HA‐MARCHF8(M10R), pCAGGS‐MARCHF9(M7R)−3×HA, and pCAGGS‐MARCHF9(M10R)−3×HA mutants were created by swamping the RING domain of MARCHF2 (aa 56–116), MARCHF8 (aa 72–133), and MARCHF9 (aa 102–162) with those from MARCHF7 (aa 544–614) or MARCHF10 (aa 689–759). MARCHF2 deletion mutants ΔN (∆1–55), ΔL (∆117–137), ΔR (∆56–116), and ΔC (∆196–246) were generated by PCR using pCAGGS‐MARCHF2‐3×HA as a template. The MARCHF8 deletion mutants ΔN (∆1–71), ΔL (∆134–156), ΔR (∆72–133), and ΔC (∆218–291) were generated by PCR using pCAGGS‐3×HA‐MARCHF8 as a template. The MARCHF9 deletion mutant ΔN (∆1–101), ΔL (∆163–184), ΔR (∆102–162), and ΔC (∆240–346) were generated by PCR using pCAGGS‐MARCHF9‐3×HA as a template. The *USP19* (GenBank XM_0 052 64825.2), *USP32* (GenBank AF533230.1), and *USP33* (GenBank AF383172.1) genes with a 3xMyc‐tag were synthesized and cloned into the pCAGGS vector by BamHI/EcoRI digestion. Expression vectors for USP32 and deletion mutants Mut‐1 (∆1–299), Mut‐2 (∆369–589), Mut‐3 (∆1–585), Mut‐4 (∆585–1604), Mut‐5 (∆734–1567), and Mut‐6 (∆1568–1604) with a 3xHA‐tag were generated by PCR using pCAGGS‐USP32‐3×Myc as a template. pCAGGS‐USP32‐C743A‐3×Myc was generated from pCAGGS‐USP32‐3×Myc by site‐directed mutagenesis.

### Western Blotting (WB)

HEK293T cells were transfected transiently using the polyethylenimine (PEI) (Polysciences, 23966‐1) as done previously.^[^ ^44^ ^]^ Before transfection, 6x10^5^ HEK293T cells were seeded in 6‐well plates in 2 mL supplemented DMEM to obtain a confluence of 60% at the time of transfection. For transfection, vector DNA was mixed with PEI and filled up with Opti‐MEM I (Gibco 31 985 070) to 200 µL. After a 20‐min incubation at room temperature, the mixture was added to the cells. After 48 h, transfected cells were lysed with 200 µL RIPA lysis buffer (Sigma, R0278) supplemented with protease inhibitors for 30 min at 4 °C. Cell lysate was cleared at 12000 × *g* for 10 min at 4 °C and mixed with a loading buffer (5×) followed by boiling at 100 °C for 10 min. Samples were then subjected to sodium dodecyl sulfate polyacrylamide gel electrophoresis (SDS‐PAGE), and transferred to polyvinylidene difluoride (PVDF) membranes (Sigma, ISEQ00010). After being blocked with 5% skimmed milk (Applygen, P1622) in TBST buffer for 1 h at room temperature, membranes were incubated with primary antibodies at 4 °C overnight. After being washed with TBST 3 times for 15 min, membranes were incubated with HRP‐conjugated secondary antibodies for 1 h at room temperature. Membranes were further washed three times with Tris‐buffered saline (20 × 10^−3^ m Tris, pH 7.4, 150 × 10^−3^ m NaCl) with 0.1% Tween 20 (Solarbio Life Sciences, T8220) (TBST) and proteins were detected by enhanced chemiluminescence (ECL) solution (Applygen, P1010).

Antibodies used include: Horseradish peroxidase (HRP)‐conjugated anti‐FLAG (Sigma, A8592, 1:8000), HRP‐conjugated anti‐HA (Sigma, H6533, 1:5000), HRP‐conjugated anti‐Actin (Proteintech, HRP‐60008, 1:10 000), HRP‐conjugated anti‐His (Proteintech, HRP‐66005, 1:8000), rabbit anti‐furin (Proteintech,18413‐1‐AP, 1:5000), mouse anti‐Myc (Cell Signaling, 2276S, 1:2000), rabbit anti‐SARS‐CoV‐2 (Sino Biological, 40590‐T62, 1:8000), mouse anti‐MERS‐CoV (Sino Biological, 40070‐MM11, 1:5000), mouse anti‐USP32 A‐10 (Santa Cruz Biotechnology, SC‐374465, 1:2000), and rabbit anti‐MARCHF2 (Abmart, PS07245S, 1:200).

### CRISPR/Cas9 Knockout and Small Interference RNA (siRNA) Silencing

To generate knockout cell lines, a pcDNA3.3 vector expressing human codon‐optimized Cas9 (Addgene, #41 815) and a pGEM‐T vector expressing specific‐small guide (sg) RNAs under a U6 promoter were co‐transfected into HEK293T cells as done previously.^[^ ^45^ ^]^ The *furin* sgRNA sequence is 5′‐GACTAAACGGGACGTGTACC‐3′. The *USP32* sgRNA sequences are 5′‐AATGGAAAGAATGCTCCACG‐3′ and 5′‐GTAAAGTCCCAGATACACTC‐3′ as reported.^[^ ^46^ ^]^ Clonal knockout cells were screened via WB. The *MARCHF2* siRNA was 5′‐GCGACAUGGUGUGUUUCCUGUUCAU‐3′/5′‐AUGAACAGGAAACACACCAUGUCGC‐3′, as reported.^[^ ^20b^ ^]^ The scrambled siRNA control was 5′‐UUCUCCGAACGUGUCACGUdTdT‐3′/5′‐ACGUGACACGUUCGGAGAAdTdT‐3′.

### Confocal Microscopy

HeLa cells were seeded in a cell culture dish with a glass bottom one day before transfection. The cells were transfected with Lipofectamine 3000 (Invitrogen, L3000‐015) according to the manufacturer's protocol. After 24 h, cells were washed with phosphate buffer saline (PBS) and fixed with 4% paraformaldehyde for 15 min at room temperature. Cells were permeabilized with 0.1% Triton X‐100 for 10 min and blocked with 10% FBS for 2 h at room temperature. Cells were then incubated with mouse anti‐FLAG (Sigma, B3111, 1:1000) or mouse anti‐HA (Sigma, H3663, 1:1000) antibody diluted in 10% FBS overnight at 4 °C. After being washed three times for 15‐min with PBS, cells were incubated with Alexa Fluor 594‐conjugated goat anti‐mouse (Invitrogen, A11012, 1:1000) antibody and 4′,6‐diamidino‐2‐phenylindole (DAPI) (Sigma, D9542, 1:1000) for 1 h at room temperature. After being washed again three times, images were acquired using a confocal microscope (ZEISS, LSM880), as done previously.^[^ ^47^ ^]^

### Immunoprecipitation

HEK293T cells were transfected in 10‐cm dishes with different expression plasmids according to experimental designs. Cells were harvested after 48 h transfection and lysed by 800 µL RIPA lysis buffer at 4 °C for 30 min. The cell lysate was cleared at 12 000 × *g* at 4 °C for 10 min. A total of 200 µL of the cytosolic fraction was collected as input and the remaining volume was incubated with anti‐FLAG M2 magnetic beads (Sigma, M8823) or anti‐HA Affinity Gel (Beyotime P2287) overnight at 4 °C. To detect furin shedding, cell culture supernatants were first cleared at 3000 × *g* for 5 min and then incubated with anti‐FLAG M2 magnetic beads overnight at 4 °C. After beads were washed with ice‐cold PBS, bead‐associated proteins were detected by W.B.

### Mass Spectrometry

HEK293T cells were transfected with the furin‐FLAG expression vector. After 48 h, cells were lysed by RIPA lysis buffer, and proteins were immunoprecipitated with anti‐FLAG M2 magnetic beads (Sigma, M8823). After being washed with PBS three times, the protein‐bound beads were mixed with loading buffer (5×) and boiled at 100 °C for 10 min. Proteins were subjected to SDS‐PAGE and detected by Coomassie blue staining. Protein gel was sent to the Laboratory of Proteomics, Institute of Biophysics, Chinese Academy of Sciences, for Nano LC‐MS/MS and database search analysis. A detailed protocol for this analysis is provided in the Supporting Information. The raw data was submitted to iProX as a project ID IPX0008971000.

### In Silico Analysis of Furin and M8 RING Domain Interaction

To analyze the protein complex of furin/M8 RING domain, ColabFold,^[^ ^25^ ^]^ a computational server equipped with AlphaFold2‐Multimer was used,^[^ ^26^ ^]^ to predict the protein–protein interactions between the full‐length furin (UNIPROT: P09958) and N‐terminal M8 (UNIPROT: P0DTC2). To define the protein–protein binding interface in the predicted complexes, the alphashape analysis was employed.^[^ ^27^ ^]^ All predicted furin/M8 complexes were analyzed, and additional details can be found in Table S1 in the Supporting Information.

### Statistical Analysis

All experiments were performed independently at least three times. SPSS Statistics Software (Version 23; IBM, Inc., New York, USA) was used for the data analysis. Quantitative values of data were expressed as mean ± standard error of measurements (SEMs) and represented by error bars.

## Conflict of Interest

The authors declare no conflict of interest.

## Author Contributions

W.S. and S.L. conducted most of the experiments with support from I.A., Y.W., and I.K.; L.T. contributed resources; B.Y. and J.L. conducted alphashape analysis; W.S., S.L., and Y.H.Z. designed experiments; Y.H.Z. wrote the manuscript with input from all authors.

## Supporting information

Supporting Information

## Data Availability

The data that support the findings of this study are available from the corresponding author upon reasonable request.
