# Supplementary material for: Furin Egress from the TGN is Regulated by Membrane‐Associated RING‐CH Finger (MARCHF) Proteins and Ubiquitin‐Specific Protease 32 (USP32) via Nondegradable K33‐Polyubiquitination
Source: Adv Sci (Weinh). 2024 Jul 19;11(35):2403732. doi: 10.1002/advs.202403732 (PMC11425283; doi:10.1002/advs.202403732)
Supplement: Supplementary file 1 — Supporting Information [file ADVS-11-2403732-s001.pdf]

ADVANCED SCIENCE
Open Access

Supporting Information

for *Adv. Sci.*, DOI 10.1002/advs.202403732

Furin Egress from the TGN is Regulated by Membrane-Associated RING-CH Finger (MARCHF) Proteins and Ubiquitin-Specific Protease 32 (USP32) via Nondegradable K33-Polyubiquitination

Wenqiang Su, Iqbal Ahmad, You Wu, Lijie Tang, Ilyas Khan, Bowei Ye, Jie Liang, Sunan Li and Yong-Hui Zheng**

Supplemental Materials

Mass Spectrometry Analysis
(provided by Laboratory of Proteomics, Institute of Biophysics, Chinese Academy of Sciences)

In-gel digestion of proteins
The entire lane of furin from IP was manually excised from the gel and digested individually as below. The protein bands were cut into small plugs, washed twice in 200 µl of distilled water for 10 min each time. The gel bands were dehydrated in 100% acetonitrile for 10 min and dried in a Speedvac for approximately 15 min. Reduction (10 mM DTT in 25 mM NH_4HCO_3 for 45 min at 56°C) and alkylation (40 mM iodoacetamide in 25 mM NH_4HCO_3 for 45 min at room temperature in the dark) were performed, followed by washing the gel plugs with 50% acetonitrile in 25 mM ammonium bicarbonate twice. The gel plugs were then dried using a Speedvac and digested with sequence-grade modified trypsin (40 ng for each band) in 25 mM NH_4HCO_3 overnight at 37 °C. The enzymatic reaction was stopped by adding formic acid to a 1% final concentration. The solution was then transferred to a sample vial for LC-MS/MS analysis

LC-MS/MS analysis
All nanoLC-MS/MS experiments were performed on a Orbitrap Exploris 480 (Thermo Scientific) equipped with an Easy n-LC 1200 HPLC system (Thermo Scientific). The peptides were loaded onto a 100 µm id×2 cm fused silica trap column packed in-house with reversed phase silica (Reprosil-Pur C18 AQ, 5 µm, Dr. Maisch GmbH) and then separated on an a 75 µm id×25 cm C18 column packed with reversed phase silica (Reprosil-Pur C18 AQ, 1.9 µm, Dr. Maisch GmbH). The peptides bound on the column were eluted with a 73-min linear gradient. The solvent A consisted of 0.1% formic acid in water solution and the solvent B consisted of 80% acetonitrile and 0.1% formic acid. The segmented gradient was 4–9% B, 3 min; 9–20% B, 22 min; 20–30% B, 20 min; 30-40% B, 15 min; 40-95% B, 3min; 95% B, 10min at a flow rate of 300 nl/min.

The MS analysis was performed with Orbitrap Exploris 480 mass spectrometer with the FAIMS Pro interface (Thermo Scientific). FAIMS separations were performed with two compensation voltage (-45 and -65). With the data-dependent acquisition mode, the MS data were acquired at a high resolution 60,000 (m/z 200) across the mass range of 350–1500 m/z. The target value was 3.00E+06 with a maximum injection time of 22 ms. Data dependent mode was selected as cycle time mode which was set as 2 seconds. The precursor ions were selected from each MS full scan with isolation width of 1.6 m/z for fragmentation in the Ion Routing Multipole with normalized collision energy of 28%. Subsequently, MS/MS spectra were acquired at resolution 15,000 at m/z 200. The target value was 7.50E+04 with a maximum injection time of 22 ms. The dynamic exclusion time was 40s. For nano electrospray ion source setting, the spray voltage was 2.0 kV; no sheath gas flow; the heated capillary temperature was 320 °C.

Protein Identification and quantification analysis
The raw data from Orbitrap Exploris 480 were analyzed with Proteome Discovery version 2.4.1.15 using Sequest HT search engine for protein identification. The Uniprot human protein database (updated on 11/2022) was used for searching the data from samples. Some important searching parameters were set as following: trypsin was selected as enzyme and two missed cleavages were allowed for searching; the mass tolerance of precursor was set as 10 ppm and the product ions tolerance was 0.02 Da.; the cysteine carbamidomethylation were specified as fixed modifications; The methionine oxidation and GlyGly addition to lysine residues were chosen as variable modifications. FDR analysis was performed with Percolator and FDR <1% was set for protein identification.

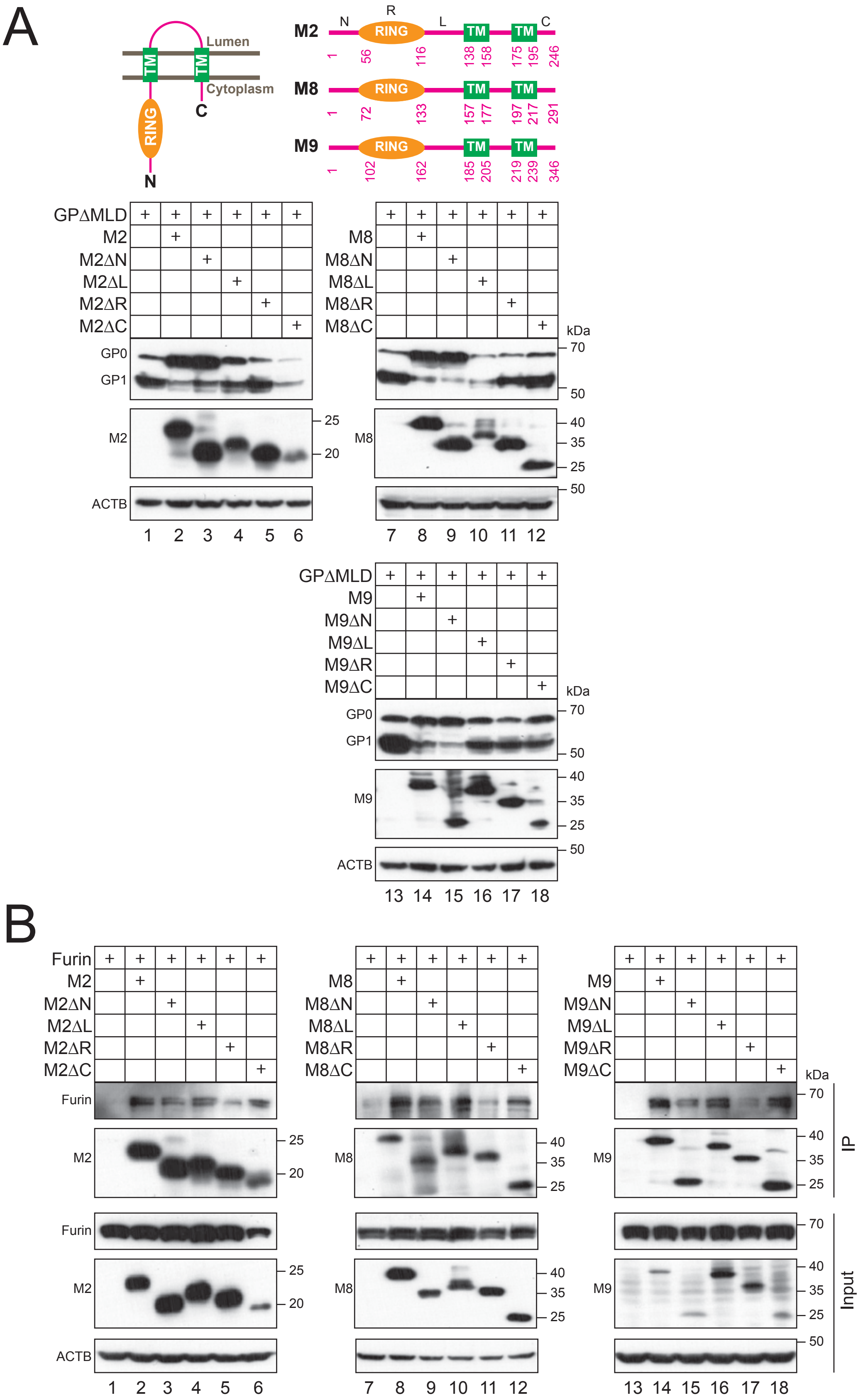

Figure S1. (A) Schematic representations of M2, M8, and M9 proteins are shown on the top. N, N-terminal domain; R, RING domain; L, linker domain; C, C-terminal domain. MARCHF mutants bearing a deletion in these domains were expressed with GPΔMLD in HEK293T cells. GP processing was determined by WB.

(B) HA-tagged MARCHF mutants were expressed with FLAG-tagged furin in HEK293T cells. Proteins were immunoprecipitated with anti-HA and analyzed by WB.

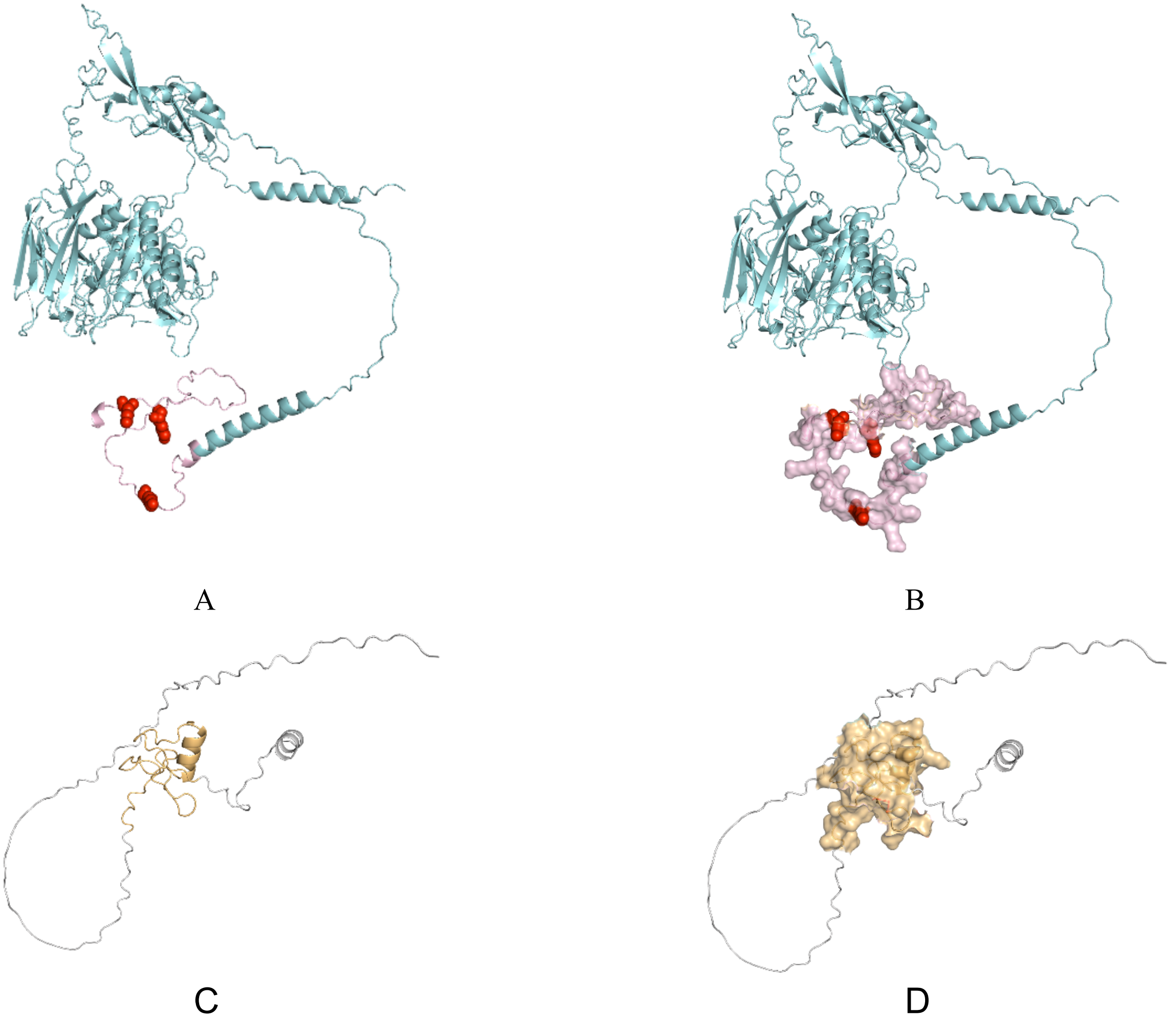

Figure S2. Structural Insights into AlphaFold2 (AF2)-predicted furin and M8 RING domain. **(A)** The AF2-predicted structure of the full-length furin protein (UNIPROT/AF2: P09958), with the CT (aa 736-794) in light pink, features a random-coil-like configuration. Lysine residues at positions 748, 760, and 789 are marked with red spheres. **(B)** A surface representation of the furin CT provides a detailed view of its spatial arrangement. **(C)** The structure of M8 N-terminal region (aa 1-157) (UNIPROT/AF2: P0DTC2), with the RING domain (aa 72-133) in light orange, consists of alpha-helices and random-coil or loop regions. This configuration is likely crucial for mediating the protein's interactions and enzymatic activities. **(D)** A surface visualization of the M8 RING domain offers a closer look at its molecular surface and potential interaction sites.

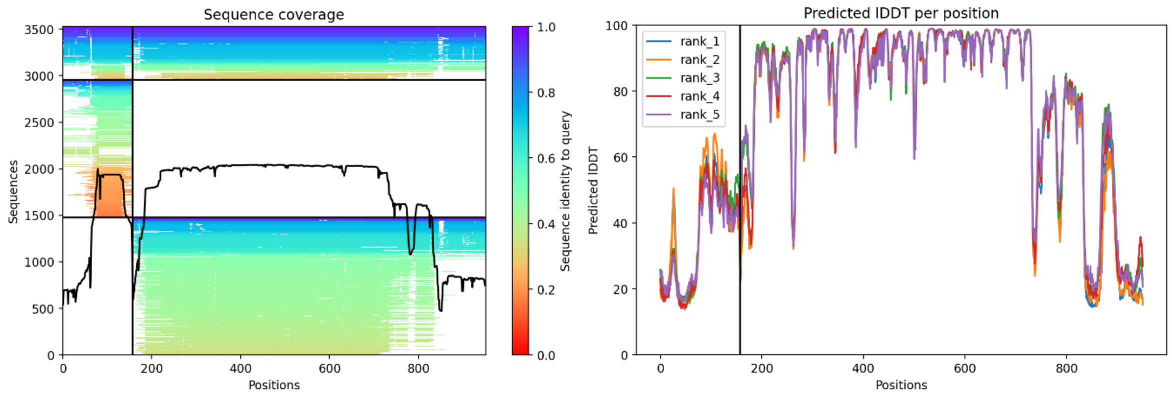

(A) Sequence coverage (B) Structure pLDDT score

Figure S3. Furin/M8 RING domain complex sequence coverage and pLDDT scores. **(A)** The M8 segment spans positions 1-157, and the furin protein is represented by positions 158-951 (corresponding to aa 1-794). Both sequences exhibit a reliable depth of multiple sequence alignment, surpassing 100. **(B)** The predicted local-distance difference test (pLDDT) score, ranging from 0 to 100, shows the residue-specific prediction confidence level. Residue with pLDDT above 70 is expected to be modeled well. The interacting region of the RING domain (aa 72 to 133) and part of the M8 CT (position 893 to 951, corresponding to aa 736-794) are approximately or exceed the threshold of 70.

	M8 RING-domain	Furin CT
Binding area	88-D, 89-D, **90-E**, 91-S, 92-P, 93-L, **94-I**, **95-T**, **96-P**, 97-C, 98-H, 104-H, 105-F, 107-H, 108-Q, 109-A, 111-L, 112-Q, 115-I, **128-Y**, 130-F, **131-I**, 132-M, **133-E**	757-I, 758-S, 759-Y, 760-K, 761-G, 762-L, 763-P, 786-A, 787-F, 788-I, 789-K, 790-D, 791-Q, 792-S

Table S1. Details of the binding interfaces of the furin/M8 RING domain complex. In bold, RING domain residues indicate direct interactions with furin K748, K760, and K789 in CT.
